# Patient-Reported Outcome Measures in Older Veterans Initiating a New Episode of Mental Health Care

**DOI:** 10.1093/geroni/igaa057.984

**Published:** 2020-12-16

**Authors:** Jenefer Jedele, Karen Austin, Sandra Resnick

**Affiliations:** 1 Veterans Health Administration, Ann Arbor, Michigan, United States; 2 Veterans Health Administration, West Haven, Connecticut, United States

## Abstract

The VA Measurement Based Care (MBC) in Mental Health (MH) Initiative supports implementing patient reported outcome measures (PROMs) for MH treatment planning and shared decision-making as a routine aspect of care. Using VHA administrative data, we identified Veterans initiating a new MH treatment episode (index encounter), i.e. prior 6-months without VHA MH encounters. We compare MH diagnoses, medications, and encounters during the 6-months from and including the index encounter by age (50-64; 65-79; 80+) between Veterans receiving 1 or more measures (PROM) to those receiving none (noPROM). The percentage of PROM Veterans decreased with age: 26.7% (50-64); 18.5% (65-79); 12.5% (80+). Consistent across age, PROM Veterans had more encounters than noPROM Veterans. In the year before treatment initiation, a smaller percentage of PROM Veterans had multiple MH diagnoses (21.0% v. 29.1%). At treatment initiation, both groups were equally likely to have multiple diagnoses (20.7% v. 20.1%); a higher percentage of the noPROM group were diagnosed with schizophrenia (3.8% v. 1.0%), bipolar (4.5% v. 2.2%), or PTSD (29.2% v. 21.8%). Substance use disorder and major depression were more prevalent in the PROM group. These patterns held across age categories. A smaller percentage of PROM Veterans had been prescribed psychotropic medication during the index encounter (32.8% v. 42.8%). For PROM Veterans, an average of 3 measures were received 1.5 months apart. The number of measures declined and the interval between measures increased with age. Potential barriers and possible efforts to target the use of PROMs with older Veteran patients are discussed.

